# Prognostics of multiple malaria episodes and nutritional status in children aged 6 to 59 months from 2013 to 2017 in Dangassa, Mali

**DOI:** 10.21203/rs.3.rs-3604955/v1

**Published:** 2023-11-15

**Authors:** Soumba Keita, Oumar Thiero, Mahamoudou Toure, Fousseyni Kane, Moussa Keita, Drissa Konate, Daouda Sanogo, Sory Ibrahim Diawara, Hamady Coulibaly, Sidibé M’Baye Thiam, Nafomon Sogoba, Mahamadou Diakite, Mali Bamako

**Affiliations:** University Clinical Research Center (UCRC), University of Sciences, Techniques and Technologies of Bamako; Department of Health Research and Education, Faculty of Medicine and Odonto Stomatology, University of Sciences, Techniques and Technologies of Bamako (USTTB), Bamako; University Clinical Research Center (UCRC), University of Sciences, Techniques and Technologies of Bamako; University Clinical Research Center (UCRC), University of Sciences, Techniques and Technologies of Bamako; University Clinical Research Center (UCRC), University of Sciences, Techniques and Technologies of Bamako; University Clinical Research Center (UCRC), University of Sciences, Techniques and Technologies of Bamako; University Clinical Research Center (UCRC), University of Sciences, Techniques and Technologies of Bamako; Malaria Research and Training Center, International Center for Excellence in Research, Faculty of Medicine and Odonto Stomatology, University of Sciences, Techniques and Technologies of Bamako (USTTB); University Clinical Research Center (UCRC), University of Sciences, Techniques and Technologies of Bamako; University Clinical Research Center (UCRC), University of Sciences, Techniques and Technologies of Bamako; Malaria Research and Training Center, International Center for Excellence in Research, Faculty of Medicine and Odonto Stomatology, University of Sciences, Techniques and Technologies of Bamako (USTTB); University Clinical Research Center (UCRC), University of Sciences, Techniques and Technologies of Bamako; University Clinical Research Center (UCRC), University of Sciences, Techniques and Technologies of Bamako

**Keywords:** Malaria, Anemia, Underweight, Nutritional status, multiple episodes

## Abstract

**Background:**

In Africa, the relationship between nutritional status and malaria remains complex and difficult to interpret in children. Understanding it is important in the development of malaria control strategies. This study evaluated the effect of nutritional status on the occurrence of multiple malaria episodes in children aged 6 to 59 months between 2013 and 2017 living in the village of Dangassa, Mali.

**Methods:**

A community-based longitudinal study was conducted using cross-sectional surveys (SSCs) at the beginning (June) and end (November) of the malaria transmission season associated with passive case detection (PCD) at the Dangassa Community Health Center. Children with asymptomatic malaria infection during cross-sectional surveys were selected and their malaria episodes followed by PCD. Palustrine indicators in person-months were estimated using an ordinal-logistic model repeated on subjects during follow-up periods.

**Results:**

The incidence rate (IR) during the period of high transmission (June to October), for 1 episode and for 2 + episodes peaked in 2013 with 65 children (IR = 95.73 per 1000 person-months) and 24 cases (IR = 35.35 per 1000 person-months), respectively. As expected, the risk of multiple episodes occurring during the period of high transmission was 3.23 compared to the period of low transmission after adjusting for other model parameters (95% CI = [2.45–4.26], p = 0.000). Children with anemia were at high risk of having multiple episodes (OR = 1.6, 95% CI [1.12–2.30], p = 0.011). However, the risk of having 2 + episodes for anemic children was higher during the period of low transmission (RR = 1.67, 95% CI [1.15–2.42], p = 0.007) compared to the period of high transmission (RR = 1.58, 95% CI [1.09–2.29], p = 0.016). The trend indicated that anemic and underweight children were significantly associated with multiple malaria episodes during the period of low transmission (p < = 0.001).

**Conclusion:**

Our results indicate that multiple episodes of malaria are significantly related to the nutritional status (anemia and underweight) of the child during the two transmission seasons and more pronounced during the dry season (period of low transmission). Further research including other malnutrition parameters will be needed to confirm our findings.

## Background

Malaria, anemia, and malnutrition are responsible for more than 50% of child mortality in the world, particularly among children under five years of age [1–3]. In Mali, malaria remains the leading cause of morbidity and mortality in children under five years of age, with an overall prevalence of 19%, according to the malaria Indicator Survey (MIS) of 2021 [4]. The Koulikoro health district is one of the third with a prevalence above the country mean prevalence (23%), following Mopti (27%), and Sikasso (26%) [4].

Besides malaria, malnutrition remains a significant proportion of morbidity and mortality among children under 5 in Mali and is mainly due to micronutrient deficiencies with high frequencies observed during the malaria transmission season (June to October) [5]. The disease affects millions of children, most of whom are die from common infections with associated immunodeficiency, even in mild forms [6]. In Mali, it affects 27% of children under 5 years of age in chronic form, 19% of whom are underweight (“low weight for age”) [7]. Being a composite indicator, underweight reflects both stunting and wasting, with no differentiation between them. Therefore, children presenting a low weight for their age are both growths stunted and wasted [8].

As one of one of the most common public health problems in the world, and especially one of the complications of malaria infection in endemic regions, anemia plays a major role in malaria morbidity and mortality [9, 10]. It is multifactorial, affecting 40% of children under 5 years of age, and half of these cases are attributed to nutritional iron deficiency [11]. More than 82% of children under 5 years in Mali suffer from anemia, with 25% suffering from mild anemia, 51% from moderate, and 6% from severe anemia [7].

Children’s nutritional status is a sensitive indicator of community health. The effects of malnutrition in children are long-lasting and extend decades beyond childhood [8]. However, the relationship between nutritional status and clinical malaria, on the one hand, and anemia on the other, remains complex and difficult to interpret. Anemia has not been found associated with the frequency of malaria episodes, but it has been demonstrated to be significantly associated with malnutrition [12]. In contrast, other studies have shown that malnutrition and anemia are associated with a higher risk of *Plasmodium* infection and infectious episodes [13, 14].

The relationship between nutritional status, anemia, and malaria episodes among children under the age of five is not well documented in Mali. Our study aimed to investigate longitudinally the influence of nutritional status on multiple episodes of clinical malaria in children aged 6–59 months asymptomatic to malaria in Dangassa, health district of Kati, from 2013 to 2017.

## Methods

### Study Area

The study was conducted in Dangassa (8° 12′ 37.253″ W and 12° 8′ 46.279″ N) which is located 75 km southwest of Bamako in the health district of Ouéléssebougou. This is a Sudan savannah eco-geographical zone with a rainy season lasting from June to October and a dry season from November to May. The dominant winds are the monsoon (rainy season) and the harmattan (dry season). Malaria transmission is seasonal in Dangassa, with 6 to 7 months of transmission per year (June to December).

### Study design

#### Cross-sectional surveys

Data on the malaria prevalence were collected through seven cross-sectional surveys: in June (at the start of the rainy season) and in October/November (at the end of the rainy season). During each survey, demographic and clinical data were collected from each participant. Microscopy (smear) was used to assess P. falciparum infection, while *HemoCue*^®^ *Hb 301* was used *to measure* hemoglobin levels. For each participant, data on individual use of LLINs and fever defined as axillary temperature ≥ 37.5°C or fever within the previous 48 hours) was collected. A blood smear was prepared for each participant during both SSCs and PCD to determine malaria parasitemia. During the SSCs, only participants with fever were tested for malaria using malaria a rapid diagnostic test RDT. Participants tested positive for malaria were provided treatment according to the policies of the Malian NMCP. We defined a malaria episode for the same participant as new when time between two subsequent infections was greater than 20 days. The number of episodes per child was classified into three categories 0,1 and 2 or more. Weight was measured using a weigh scale. ENA For Smart software was used to calculate scores (z-scores) based on the national results at the WHO reference population. Malnutrition indicators were determined using a standard deviation of −2 standard deviations or Z-score. The weight-for-age (WAZ) of children was classified in two categories, Z-score according to WHO growth standards references for children aged zero to five years 16. Score ≤ −2 standard deviation (SD) were defined as underweight, while score > −2 standard were defined as normal from the reference mean. Anemia was classified into two categories: Anemia: Hb level < 11 g/dl; No anemia: Hb level ≥ 11 g/dl 18.

### Passive case detection (PCD)

Through community health based passive surveillance a physician and a biologist followed the cohort participants for malaria incidence from February 2013 to March 2017. During this follow-up, data on anemia and malnutrition were collected in the same way as during the cross-sectional surveys. Study participants with fever were invited to the health center (suspected malaria cases) and tested using RDT and microscopy. All RDT positive cases received free antimalarial treatment according to national policy by the local health staff

### Laboratory methods

Blood films were stained with 10% Giemsa and examined under a 100X oil immersion objective lens of a light microscope. Asexual parasites were estimated based on the number of parasitized red blood cells per 300 white blood cells multiplied by 25, assuming an average white blood cell count of 7500/μl. A minimum of 100 fields were examined before a thick drop was considered negative. by Two different experienced microscopists read each smear separately. RDTs (*BIOLANE*^®^) for *P. falciparum* infection were performed according to the manufacturer’s instructions. RDTs used were those currently recommended by the NMCP of Mali. Hemoglobin concentration was measured in venous blood using HemoCue 301. And anemia was defined as a hemoglobin level < 11 g/dl (16).

### Statistical methods

Microsoft Access 2019 was used for database management and to track the malaria episode using PCD data between two SSCs. The data and graphical analysis were done using SAS version 9.2 and SPSS version 28.

For the malaria indicators in person-months, a repeated ordinal-logistic regression model. The Generalized Estimating Equation (GEE) with the hybrid method (in which Fisher scoring iterations are performed before switching to the Newton-Raphson method) for the parameter’s estimations and maximum likelihood for the algorithm convergence was applied on the model. The working correlation matrix has shown an exchangeable structure. The repeated subject effect was the children study ID with the intrasubject effect as the follow-up period. The convergence criteria were satisfied, and the data fit well the model. The children exposure times within each transmission period were computed as the offset. The outcome variable for the model was the episodes numbers with three categories levels 0,1 or 2 + episodes. Five risk factors: age groups, gender, anemia status, weight for age (WAZ) and transmission period) were used. From the model, the predict Incidence rate (IR), odd ratio (OR) and/or the relative risk (RR) for having higher malaria-episodes numbers were estimated.

## Results

Seven SSCs, from 2013 (February), 2014 (June, October), 2015 (June, November) and 2016 (June, December), were carry-out during which 199,107,233,151,324,232 and 154 children were respectively enrolled at the beginning of each of the malaria-transmission-periods. The prevalence of clinical malaria was 15.6% in 2013, 15.0% vs 16.7%, 8.6% vs 13.6%, and 6.9% vs 13.6% respectively at the start and end of transmission in 2014, 2015, and 2016. Table 1.

Using the PCD data, the unadjusted comparison of no zero percentage for malaria episode numbers by each risk factor and by transmission period indicated a significant difference between sex in 2013 (February to June), in WAZ status in 2014 (June to October) and in Anemia status in 2015 (June to November) (all p < 0.05). There was no significant difference elsewhere. Table 2a

The peak of incidence risk (IR) for one (IR = 95.73 in 1000 person-months, n = 65) and 2+ (IR = 35.35 in 1000 person-months, n = 24) episodes was observed in 2013 during the high transmission period (June to October). IR decreased to zero cases at the end of the study. The lowest IR for 1 episode and 2 + episodes were observed in the second half time of the study follow-up period in 2016 (June – December) with 9 cases (IR = 6.78 in 1000 person-months) and 5 cases (IR = 3.77 in 1000 persons-months), respectively. Table 2b

**Figure 3:**
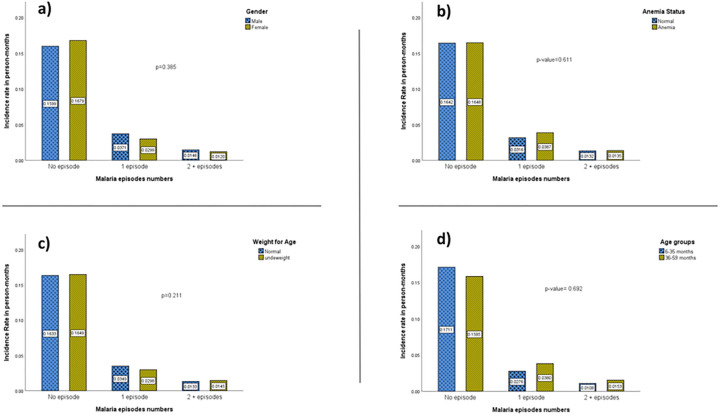
Univariate comparison for having higher episode numbers by risk factors percentage within each Malaria episodes numbers during the follow-up’s time in person year.

The univariate analysis of risk factors between age groups indicated that children 36–59 months were more likely to have higher numbers of malaria episodes than 6–35 months old. Males were more likely to experience a higher number of multiple malaria episodes as well as anemia compared to females. However, none of the differences was significant (all p > 0.05). Figure 3, a, b, c and d.

After adjusting for all other parameters, the odds ratio (OR) for having a higher number of malaria episodes during the high transmission period was higher than in the low transmission period (OR = 3.23, 95% CI = [2.45–4.26], p = 0.000). The children with anemia were more likely to have higher numbers of malaria episodes (OR = 1.6, 95% CI [1.12–2.30], p = 0.011) than those with normal hemoglobin levels. Table 3

**Figure 4:**
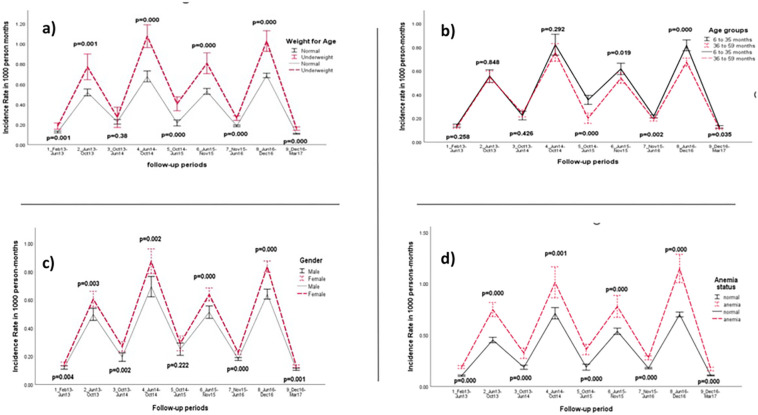
Trend of the predicted Incidence Rate in person-months of children under 5 years for Malaria 2+ episodes by (a) Age’s, (b) Gender’s, (c) Anemia’s and (d) Weight for Age’s groups in Dangassa from 2013 to 2017.

During Low and High transmission periods, the trends of the predicted IR in person-months among the categories of each risk factor are shown in Figs. 4a, b, c, and d. The trend indicated a significant difference between Gender, WAZ, and Anemia status during the high transmission period. However, during the low transmission period, anemic and underweight children were more likely to have 2 + malaria episodes (all p < = 0.001) except for underweight children in the follow-up period from October 2013 to June 2014, (p = 0.38).

In contrast, the Relative Risk of two or more episodes of anemia in low transmission periods (RR = 1.67, 95%CI [1.15–2.42], p = 0.007) was slightly higher than in high transmission periods (RR = 1.58, 95%CI [1.09–2.29], p = 0.016). Table 4

## Discussion

In endemic countries, malaria is a major public health problem, particularly in low-income countries [15]. Despite the complexity of the interaction between malaria, anemia, and nutritional status [1], understanding their relationship is vital for the development of strategies that will reduce child morbidity and mortality. Using repeated cross-sectional surveys and passive case detection at a community health center for five successive years, our study examined the relationship between anemia, underweight, and multiple malaria episodes among children under the age of five in Dangassa.

Based on our baseline descriptive data we found that symptomatic malaria cases varied between SSCs. The number of cases was higher at the end of the rainy season than at the beginning. In malaria-endemic areas, the parasite density is usually higher at the end than the start of the rainy season [16]. During this period, the entomological inoculation rate is particularly high [17]. Toure et al. (2016) in Sélingué, Mali [18] showed that malaria is seasonal in Mali, with a peak at the end of the rainy season. However, in 2013 a single SSCs was carried out in February after the enrolment of the study participants in 2012. Despite the survey being conducted during the dry season, the prevalence of symptomatic malaria was 15.6%, which is quite high compared with the prevalence at the start of the transmission season, i.e., 6.9–15%. Since Dangassa is a riverside village where transmission is particularly prolonged (5 to 6 months), high spatial and temporal clustering of *P. falciparum* infection during the dry season has been demonstrated [19].

As a baseline, asymptomatic children were selected at each SSCs as a baseline, and this allowed the number of malaria episodes were counted and categorized as 0, 1 episode and 2 + episodes for each child. The percentage of zero malaria episodes in the follow-up period from June to November 2015 indicated a significant difference according to anemia status. Anemia is a major cause of malaria in tropical areas and is also an additional indicator for monitoring the burden of malaria in the community [20], this finding despite the high malaria transmission period could be attributed to the introduction of seasonal malaria chemoprevention in Dangassa in 2015 [21]. Indeed, studies have shown a positive impact of seasonal malaria chemoprevention (SMC) on reducing malaria transmission and malaria indicators such as anemia in children in West Sahelian Africa [22, 23].

The incidence peak in our study was observed in 2013 during the period of high transmission (June to October), with respectively 65 children ((IR = 95.73 in 1000 person-months) for multiple episodes and 24 cases (IR = 35.35 in 1000 person-months) for those with 1 episode. Most malaria cases detected by PCD in Dangassa are symptomatic and occur during the five- to six-month transmission season (June to November) [24]. Also, Ateba F et al in 2020, reported a higher number of cases during the period when SMC was not distributed compared with the period when SMC was implemented in the same area [25].

However, the frequency of malaria episodes decreased significantly between 2013 and 2017 in our cohort. As a result, the intensification of malaria control strategies in Dangassa, including the distribution of LLINs and the introduction of SMC, have significantly reduced the burden of malaria in Dangassa [26]; Community sensitization and participation in the study; Qualified staff deployed within the framework of the International Centre of Excellence for Malaria Research in West Africa (ICEMR-1) project, the basis of this study, for the passive case detection of malaria at the health center; Health center use by residents.

According to our results, underweight is associated with a higher number of episodes compared to normal children 10% error (OR = 1.46, 95% CI [0.95–2.23], p = 0.084). Almeida M. et al in 2015 [27], in a rural community in the Amazon region, demonstrated that malaria-induced anorexia and vomiting during the acute phase of the disease, associated with insufficient micronutrients, in children under five years of age in a specific endemic context, could lead to a delay in the child’s physical development after several episodes of malaria.

The adjusted model showed a significant association between multiple episodes in anemic and normal children (OR = 1.6, 95%CI [1.12–2.30], p = 0.011). Thus, in high transmission areas such as Dangassa, where the disease is prevalent, many young children are anemic, and people infected with the disease may receive a single bite each day, exposing them to repeated episodes of the disease. Consequently, in these contexts, severe malarial anemia (hemoglobin < 7g/dl) [28], in young children, a blood transfusion is required, which characterizes a compromised rapid recovery of the anemia, since malaria infection causes hemolysis of parasitized and non-parasitized erythrocytes, and dyserythropoiesis of the bone marrow [29].

The occurrence of multiple episodes was significantly associated with anemic and underweight children during the low transmission period. Anemia has a complex etiology, including single- and multifactorial causality [30]. Thus, the high occurrence of malaria in Dangassa during the low transmission period in anemic and underweight children is associated probably through protein-energy and vitamin deficiencies. from the Democratic Republic of Congo, Aimée M and al in 2018, reported that iron deficiency can negatively affect children’s weight growth [31]. Mariken and al in 2021, measuring the association between nutritional status and malaria incidence in young children before the malaria transmission season, found that, underweight was associated with a higher incidence of clinical malaria in Burkina Faso [32].

We found that the risk of having multiple malaria episodes during the high transmission period was 3.23 times higher than during the period of low transmission (CI [2.45–4.26]; p = 0.000). Although the number of multiple malaria episodes increase following exposure to malaria transmission, the rainfall, which is low during periods of low transmission and high during periods of high transmission, is an important factor in the dynamics of malaria transmission. Toure M et al (2016) reported the same results and showed a clear seasonal pattern, with a reduced below 50% malaria incidence during the dry season.

The study has the limitation that, because the data were analyzed secondary, some anthropometric parameters needed to determine children’s nutritional status were not collected. Consequently, some variables related to these indices were not able to be incorporated into our data analysis.

## Conclusion

The presence of multiple malaria episodes was significantly associated with nutritional status (anemia and low weight-for-age) in children during both high and low transmission periods. However, this relationship was more pronounced during the dry season (“low transmission” period). A further study including more parameters are necessary to support this hypothesis.

## Figures and Tables

**Figure 1:**
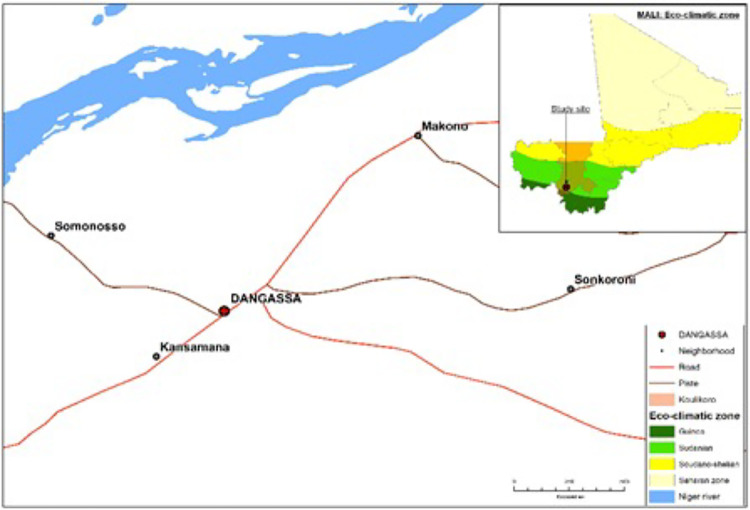
Study site of Dangassa, Oueléssébougou health district, Kati.

**Figure 2:**
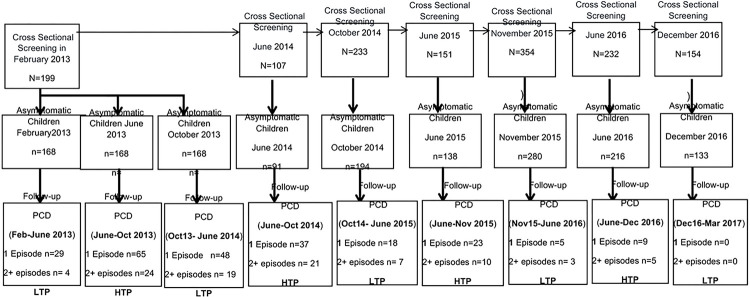
Follow up using Passive Case Detection (PCD) of children under 5 years for multiples malarial episodes during Low and High Transmission Periods (LTP and HTP) in Dangassa, from 2013 to 2017

**Table 1:** Demographics and clinicals characteristics at the cross-sectional survey for children under 5 years from 2013 to 2016 in Dangassa

Age groups in months	Feb.2013 n (%)	Jun 2014 n (%)	Oct 2014 n (%)	June 2015 n (%)	Nov. 2015 n (%)	Jun 2016 n (%)	Dec. 2016 n (%)
	N = 199	N = 107	N = 233	N = 151	N = 324	N = 232	N = 154
Less than 36	47 (23.6)	1 (0.9)	39 (16.7)	32 (21.2)	110 (34.0)	64 (27.6)	36 (23.4)
36 – less 59	152 (76.4)	106 (99.1)	194 (83.3)	119 (78.8)	214 (66.0)	168 (72.4)	118 (76.6)
**Sex**							
Male	97 (48.7)	58 (54.2)	123 (52.8)	82 (54.3)	156 (49.4)	110 (48.2)	72 (42.9)
Female	102 (51.3)	49 (45.8)	110 (47.2)	69 (45.7)	160 (50.6)	118 (51.8)	80 (57.1)
**Anemia Status**							
Normal	76 (38.4)	22 (20.6)	82 (30.7)	21 (13.9)	81 (25.2)	18 (7.8)	37 (24.2)
Anemia	122 (61.6)	85 (79.4)	150 (69.3)	130 (86.1)	241 (74.8)	212 (92.2)	116 (75.8)
**Weight for age (WAZ)**							
Normal	168 (85.3)	86 (80.4)	169 (73.5)	122 (82.4)	252 (81.6)	188 (84.7)	114 (77.6)
Underweight	29 (14.7)	21 (19.6)	61 (26.5)	26 (17.6)	57 (18.4)	34 (15.3)	33 (22.4)
**Malaria infection**							
No	104 (52.3)	43 (40.2)	121 (51.9)	118 (78.1)	137 (42.3)	188 (81.0)	116 (75.3)
**Yes**	95 (47.7)	64 (59.8)	112 (48.1)	33 (21.9)	187 (57.7)	44 (19.0)	38 (24.7)
**Fever**							
No	156 (78.4)	83 (77.6)	168 (72.1)	105 (69.5)	264 (81.5)	185 (79.7)	104 (67.5
**Yes**	43 (21.6)	24 (22.4)	65 (27.9)	46 (30.5)	60 (18.5)	47 (20.3))	50 (32.5)
**Malaria cases**							
Asymptomatic	168 (84.4)	91 (85.0)	194(83.3)	138 (91.4)	280 (86.4)	216 (93.1)	133 (86.4)
Clinical Malaria	31 (15.6)	16 (15.0)	39 (16.7)	13 (8 .6)	44 (13.6)	16 (6.9)	21 (13.6)

**Table 2a:** Comparison of numbers of cases for malaria multiples episodes by risk factors and by follow-up periods for children under 5 years from 2013 to 2017 in Dangassa

Follow-up periods	Episodes numbers	Age groups in months			Gender			Anemia Status			WAZ		
		6-35	36-59	p-value	Male	Female	p-value	Normal	Anemia	p-value	Normal	Underweight	p-value
		Cases n	Cases n		Cases n	Cases n		Cases n	Cases n		Cases n	Cases n	
Feb-Jun 2013	**0**	67	68	0.58	64	71	0.04*	88	46	0.76	119	15	0.09
	**1**	14	15		12	17		20	9		23	6	
	**2 +**	3	1		4	0		2	2		2	2	
Jun-Oct. 2013	**0**	34	45	0.18	34	45	0.40	52	26	0.93	69	9	0.73
	**1**	38	27		32	33		43	22		55	10	
	**2 +**	12	12		14	10		15	9		20	4	
Oct. 2013-Jun 2014	**0**	45	56	0.19	46	55	0.76	65	35	0.40	89	11	0.27
	**1**	29	19		25	23		30	18		38	10	
	**2 +**	10	9		9	10		15	4		17	2	
Jun-Oct. 2014	**0**	17	45	0.11	32	30	0.63	47	15	0.62	43	19	0.04*
	**1**	11	26		22	15		29	8		33	4	
	**2 +**	11	10		10	11		18	3		14	7	
Oct. 2014-Jun. 2015	**0**	44	29	0.00	39	34	0.33	37	34	0.21	48	22	0.30
	**1**	0	18		11	7		12	6		15	3	
	**2 +**	0	7		2	5		2	5		6	1	
Jun-Nov 2015	**0**	93	75	0.00	79	85	0.12	138	30	0.03*	133	28	0.51
	**1**	0	23		16	7		22	1		21	2	
	**2 +**	0	10		4	6		10	0		8	2	
Nov. 2015-Jun. 2016	**0**	108	101	0.00	99	106	0.10	157	50	0.43	156	43	0.15
	**1**	0	5		3	2		4	1		5	0	
	**2 +**	0	3		3	0		3	0		3	0	
Jun-Dec. 2016	**0**	103	99	0.00	93	105	0.17	184	17	0.62	164	30	0.38
	**1**	0	9		6	3		8	1		7	2	
	**2 +**	0	5		4	1		5	0		5	0	
Dec. 2016-Mar 2017	**0**	59	74		61	70		104	28		100	28	

**Table 2b:** Incidence rate in 1000 person months of malaria episodes numbers for children under 5 years from 2013 to 2017 in Dangassa

Follow- up Periods	Episodes Numbers					
	1			2+		
	nb. Cases	nb. person months	Incidence Rate	nb. Cases	nb. person months	Incidence Rate
Feb-Jun 2013	29	521	55.66	4	521	7.68
Jun-Oct 2013	65	679	95.73	24	679	35.35
Oct13-Jun 2014	48	916	52.40	19	916	20.74
Jun-Oct 2014	37	706	52.41	21	706	29.75
Oct14-Jun 2015	18	547	32.91	7	547	12.80
Jun-Nov 2015	23	923	24.92	10	923	10.83
Nov15-Jun 2016	5	1058	4.73	3	1058	2.84
Jun-Dec 2016	9	1327	6.78	5	1327	3.77
Dec16-Mar 2017	0	389	0.00	0	389	0.00

**Table 3:** Univariate and Multivariate Odds Ratio by risk factors of having higher number of malaria episodes in person years using Repeated Ordinal logistics Regression model (Repeated Proportional odds Model) with time of exposure as the offset.

	GEE: Repeated Proportional odds Model					
	Univariate			Multivariate		
	OR	95%CI OR	p-value	OR	95%CI OR	p-value
**Age Groups** (months)						
36–59 vs. 6–35	0.91	0.58–1.43	0.692	0.97	0.64–1.47	0.890
**Gender**						
Female vs. Male	1.31	0.71–2.43	0.385	1.29	0.78–2.15	0.336
**Anemia status**						
Anemia vs. Normal	1.13	0.71–1.78	0.611	1.60	1.12–2.30	0.011
**Weight for age (WAZ)**						
Underweight Vs. Normal	1.38	0.84–2.26	0.211	1.46	0.95–2.23	0.084
**Transmission period**						
High Vs. Low	3.01	2.37–3.84	0.0001	3.23	2.45–4.26	0.0001

**Table 4:** Predicted Incidence Rate in 1000-person-months and Relative Risk by transmission periods (low and High) and by risk factors of Malaria multiples episodes (2 + episodes) among children under 5 years from 2013 to 2017 in Dangassa.

	Low Transmission				High Transmission			
	Incidence Rate	RR	CI 95%	p-value	Incidence Rate	RR	CI 95%	p-value
**Age Group (**months**)**								
36–59	0.18	0.90	0.60–1.37	0.616	0.69	1.09	0.72–1.65	0.682
6–35 (ref)	0.20				0.63			
**Gender**								
Female	0.21	1.23	0.74–2.05	0.426	0.73	1.25	0.75–2.08	0.391
Male (ref)	0.17				0.58			
**Anemia Status**								
Anemia – yes	0.27	1.8	1.24–2.61	0.002	0.86	1.41	0.97–2.05	0.071
Anemia-No (ref)	0.15				0.61			
**Weight for age (WAZ)**								
Underweight	0.26	1.53	0.99–2.35	0.053	0.32	1.52	0.99–2.34	0.057
Normal (ref)	0.17				0.21			

## List Of Abbreviations

CI: Confident Interval
SSCs: Cross Sectional surveys
GEE: Generalized Estimating Equation
Hb: Hemoglobin
HTP: High Transmission Period
ICEMR-WA: International Centre of Excellence for Malaria Research in West Africa
IR: Incidence rate
LLINs: Insecticide-Impregnated Mosquito Nets
LTP: Low Transmission Period
MIS: Malaria Indicator Survey
NMCP: National Malaria Control Program
OR: Odd ratio
PCD: Passive Case Detection
RDT: Rapid Diagnostic Test
RR: Relative Risk
SD: Standard Deviation
SMC: Seasonal Malaria Chemoprevention
WAZ: Weight-for-Age
WHO: World Health Organization

## References

[1] Das D, Grais RF, Okiro EA, Stepniewska K, Mansoor R, van der Kam S, Terlouw DJ, Tarning J, Barnes KI, Guerin PJ: Complex interactions between malaria and malnutrition: a systematic literature review. BMC Med 2018, 16:186. 30371344 10.1186/s12916-018-1177-5 PMC6205776

[2] Ehrhardt S, Burchard GD, Mantel C, Cramer JP, Kaiser S, Kubo M, Otchwemah RN, Bienzle U, Mockenhaupt FP: Malaria, anemia, and malnutrition in african children--defining intervention priorities. J Infect Dis 2006, 194:108–114. 16741889 10.1086/504688

[3] Olofin I, McDonald CM, Ezzati M, Flaxman S, Black RE, Fawzi WW, Caulfield LE, Danaei G, Nutrition Impact Model S: Associations of suboptimal growth with all-cause and cause-specific mortality in children under five years: a pooled analysis of ten prospective studies. PLoS One 2013, 8:e64636. 23734210 10.1371/journal.pone.0064636 PMC3667136

[4] Institut National de la Statistique Institut , Programme National de Lutte contre le Paludisme , ICF: Malaria Indicator Survey in Mali 2021. Bamako, Mali et Rockville, Maryland, USA: INSTAT, PNLP et ICF; 2022.

[5] Teh RN, Sumbele IUN, Meduke DN, Ojong ST, Kimbi HK: Malaria parasitaemia, anaemia and malnutrition in children less than 15 years residing in different altitudes along the slope of Mount Cameroon: prevalence, intensity and risk factors. Malar J 2018, 17:336. 30249261 10.1186/s12936-018-2492-1 PMC6154899

[6] Rytter MJ, Kolte L, Briend A, Friis H, Christensen VB: The immune system in children with malnutrition--a systematic review. PLoS One 2014, 9:e105017. 25153531 10.1371/journal.pone.0105017 PMC4143239

[7] Institut National de la Statistique - INSTAT, Cellule de Planification et de Statistique Secteur Santé-Développement, ICF: Mali Demographic and Health Survey 2018. Bamako, Mali: INSTAT/CPS/SS-DS-PF and ICF; 2019.

[8] Nigatu G, Assefa Woreta S, Akalu TY, Yenit MK: Prevalence and associated factors of underweight among children 6–59 months of age in Takusa district, Northwest Ethiopia. Int J Equity Health 2018, 17:106. 30041638 10.1186/s12939-018-0816-y PMC6057034

[9] Green HK, Sousa-Figueiredo JC, Basanez MG, Betson M, Kabatereine NB, Fenwick A, Stothard JR: Anaemia in Ugandan preschool-aged children: the relative contribution of intestinal parasites and malaria. Parasitology 2011, 138:1534–1545. 21819635 10.1017/S0031182011001016

[10] Morakinyo OM, Balogun FM, Fagbamigbe AF: Housing type and risk of malaria among under-five children in Nigeria: evidence from the malaria indicator survey. Malar J 2018, 17:311. 30153834 10.1186/s12936-018-2463-6 PMC6114872

[11] WHO: Anaemia. 2023.

[12] Muller O, Traore C, Jahn A, Becher H: Severe anaemia in west African children: malaria or malnutrition? Lancet 2003, 361:86–87. 10.1016/S0140-6736(03)12154-X 12517511

[13] Sumbele IU, Kimbi HK, Ndamukong-Nyanga JL, Nweboh M, Anchang-Kimbi JK, Lum E, Nana Y, Ndamukong KK, Lehman LG: Malarial anaemia and anaemia severity in apparently healthy primary school children in urban and rural settings in the Mount Cameroon area: cross sectional survey. PLoS One 2015, 10:e0123549. 25893500 10.1371/journal.pone.0123549 PMC4403990

[14] Yadav CP, Hussain SSA, Pasi S, Sharma S, Bharti PK, Rahi M, Sharma A: Linkages between malaria and malnutrition in co-endemic regions of India. BMJ Glob Health 2023, 8. 10.1136/bmjgh-2022-010781 PMC9853155 36653068

[15] WHO: Malaria. 2023.

[16] Mayengue PI, Kouhounina Batsimba D, Niama RF, Ibara Ottia R, Malonga-Massanga A, Fila-Fila GPU, Ahombo G, Kobawila SC, Parra HJ: Variation of prevalence of malaria, parasite density and the multiplicity of Plasmodium falciparum infection throughout the year at three different health centers in Brazzaville, Republic of Congo. BMC Infect Dis 2020, 20:190. 32131754 10.1186/s12879-020-4913-3 PMC7057455

[17] PMI: Vectorlink Mali annual entomological monitoring report. 2019.

[18] Toure M, Sanogo D, Dembele S, Diawara SI, Oppfeldt K, Schioler KL, Haidara DB, Traore SF, Alifrangis M, Konradsen F, Doumbia S: Seasonality and shift in age-specific malaria prevalence and incidence in Binko and Carriere villages close to the lake in Selingue, Mali. Malar J 2016, 15:219. 27091046 10.1186/s12936-016-1251-4 PMC4836195

[19] Shaffer JG, Toure MB, Sogoba N, Doumbia SO, Gomis JF, Ndiaye M, Ndiaye D, Diarra A, Abubakar I, Ahmad A, : Clustering of asymptomatic Plasmodium falciparum infection and the effectiveness of targeted malaria control measures. Malar J 2020, 19:33. 31964378 10.1186/s12936-019-3063-9 PMC6975028

[20] White NJ: Anaemia and malaria. Malar J 2018, 17:371. 30340592 10.1186/s12936-018-2509-9 PMC6194647

[21] Konate D, Diawara SI, Toure M, Diakite SAS, Guindo A, Traore K, Diarra A, Keita B, Thiam S, Keita M, : Effect of routine seasonal malaria chemoprevention on malaria trends in children under 5 years in Dangassa, Mali. Malar J 2020, 19:137. 32252774 10.1186/s12936-020-03202-y PMC7137428

[22] Druetz T: Evaluation of direct and indirect effects of seasonal malaria chemoprevention in Mali. Sci Rep 2018, 8:8104. 29802375 10.1038/s41598-018-26474-6 PMC5970148

[23] Diawara F, Steinhardt LC, Mahamar A, Traore T, Kone DT, Diawara H, Kamate B, Kone D, Diallo M, Sadou A, : Measuring the impact of seasonal malaria chemoprevention as part of routine malaria control in Kita, Mali. Malar J 2017, 16:325. 28797263 10.1186/s12936-017-1974-x PMC5553795

[24] Doumbia S, Toure M, Sogoba N, Alifrangis M, Diakite M, Diarra A, Keita M, Konate D, Diawara SI, Thiam SM, : The West Africa ICEMR Partnerships for Guiding Policy to Improve the Malaria Prevention and Control. Am J Trop Med Hyg 2022, 107:84–89. 36228908 10.4269/ajtmh.21-1330 PMC9662222

[25] Ateba FF, Febrero-Bande M, Sagara I, Sogoba N, Toure M, Sanogo D, Diarra A, Magdalene Ngitah A, Winch PJ, Shaffer JG, : Predicting Malaria Transmission Dynamics in Dangassa, Mali: A Novel Approach Using Functional Generalized Additive Models. Int J Environ Res Public Health 2020, 17. 10.3390/ijerph17176339 PMC7504016 32878174

[26] Doumbia S, Sogoba N, Diakite M, Toure M, Keita M, Konate D, Diawara SI, Diarra A, Sanogo D, Kane F, : A Decade of Progress Accelerating Malaria Control in Mali: Evidence from the West Africa International Center of Excellence for Malaria Research. Am J Trop Med Hyg 2022, 107:75–83. 36228923 10.4269/ajtmh.21-1309 PMC9662231

[27] Alexandre MA, Benzecry SG, Siqueira AM, Vitor-Silva S, Melo GC, Monteiro WM, Leite HP, Lacerda MV, Alecrim M: The association between nutritional status and malaria in children from a rural community in the Amazonian region: a longitudinal study. PLoS Negl Trop Dis 2015, 9:e0003743. 25928774 10.1371/journal.pntd.0003743 PMC4415998

[28] Dejene BE, Abuhay TM, Bogale DS: Predicting the level of anemia among Ethiopian pregnant women using homogeneous ensemble machine learning algorithm. BMC Med Inform Decis Mak 2022, 22:247. 36138398 10.1186/s12911-022-01992-6 PMC9494842

[29] Mwaiswelo RO, Mmbando BP, Chacky F, Molteni F, Mohamed A, Lazaro S, Mkalla SF, Samuel B, Ngasala B: Malaria infection and anemia status in under-five children from Southern Tanzania where seasonal malaria chemoprevention is being implemented. PLoS One 2021, 16:e0260785. 34855878 10.1371/journal.pone.0260785 PMC8638878

[30] Safiri S, Kolahi AA, Noori M, Nejadghaderi SA, Karamzad N, Bragazzi NL, Sullman MJM, Abdollahi M, Collins GS, Kaufman JS, Grieger JA: Burden of anemia and its underlying causes in 204 countries and territories, 1990–2019: results from the Global Burden of Disease Study 2019. J Hematol Oncol 2021, 14:185. 34736513 10.1186/s13045-021-01202-2 PMC8567696

[31] Musimwa AM, Kitoko HT, Wakamb GK, Okitotsho SW, Numbi OL: [Serum iron concentration in malnourished children from an urban and rural area in Democratic Republic of the Congo]. Pan Afr Med J 2018, 31:55. 30923600 10.11604/pamj.2018.31.55.16089 PMC6431419

[32] de Wit M, Cairns M, Compaore YD, Sagara I, Kuepfer I, Zongo I, Barry A, Diarra M, Tapily A, Coumare S, : Nutritional status in young children prior to the malaria transmission season in Burkina Faso and Mali, and its impact on the incidence of clinical malaria. Malar J 2021, 20:274. 34158054 10.1186/s12936-021-03802-2 PMC8220741

